# Facework and trust in facilitating health-focused housing interventions

**DOI:** 10.1371/journal.pone.0176074

**Published:** 2017-04-20

**Authors:** Dominic Aitken, Philip Hodgson, Glenda Cook, Allison Lawson

**Affiliations:** 1 School of Architecture, Planning & Landscape, Newcastle University, Newcastle-upon-Tyne, United Kingdom; 2 Department of Nursing, Midwifery and Health, Northumbria University, Newcastle-upon-Tyne, United Kingdom; Western Sydney University, AUSTRALIA

## Abstract

The link between housing and health is of increasing importance in the UK policy and practice context, in which poor housing is often accepted as a social determinant of poor health. Service users’ experiences of, and outcomes from, a British information, advice, support and guidance service focused on the relationship between housing problems and health issues were explored. This service facilitates home improvements for privately housed residents with housing issues exacerbating or causing health problems. In-depth interviews with occupants of 15 households which received the service were completed. The findings highlighted three key themes: the need for participants’ person-home fit to reflect and adapt to any degeneration in health conditions; the facilitation of knowledge and access to housing and other support available to them; and perceived positive health and wellbeing outcomes from the receipt of personalised advice and support. The delivery of these outcomes by a named officer of the service team, and the relationship this developed with the service user, are considered within the context of facework, whereby they became a trusted source of guidance in navigating a complex system of assistance. These findings add to the growing knowledge base on housing and health, and also highlight the critical importance of a facework approach in allowing service users to navigate complex systems in order to achieve beneficial outcomes.

## Introduction

The relationship between housing and health has recently been receiving considerable international attention from academics and policymakers [1], with research conducted in countries such as the USA [2], New Zealand [3], Singapore [4] and Ireland [5]. Evidence on the subject can be found for a wide range of settings [6–7] and populations [5; 8]. The UK has been a major focus for research in this area [1]. In the UK there is a great emphasis on understanding the impact of poor housing [9]; particularly as a social determinant of poor health [10–14]. There is also recognition that poor housing is placing a financial burden on UK health services, with an estimated cost of £1.4 to £2.5 billion [15].

The UK’s Care Act 2014 placed a duty on local authorities to integrate services including housing which have the potential to improve health and wellbeing through the prevention, reduction or delay of care needs [16]. A 2014 Memorandum of Understanding set out support for greater focus on the promotion of health through home improvement by national organisations and government departments [17]. This policy framework addresses the risks poor housing poses to vulnerable groups through cold and fuel poverty, hazards, barriers to mobility, accessibility issues and security concerns, and the impact that tackling poor housing can have on health and care needs, hospital (re)admissions and patient recovery [17].

Against this backdrop some local areas have developed innovative approaches to the integration of housing, health and adult social care services [18]. This paper explores clients’ experiences and outcomes from North Tyneside’s Safe and Healthy Homes (SHH) service. This service’s team provides information, advice, support and guidance to residents living in private rented or owner-occupied accommodation who have a housing issue which affects or has the potential to affect their physical or mental health. The findings revealed that before the involvement of SHH, participants had limited knowledge and understanding of, and trust in, organisations and services that could assist them in rectifying housing issues affecting their health and wellbeing. The study adds to the understanding of health-focussed housing interventions by exploring the operation of an ongoing advice service, where previous research has tended to focus upon one-off interventions [19–21]. The contribution of this study is the demonstration of how access to health-focussed housing interventions is critically dependent upon ‘facework’ by officers who can become a trusted source of guidance in navigating a complex system of assistance and services [22].

## Housing and health

In addition to being influenced by individual lifestyle, constitutional and social factors, health is determined by a variety of living and working conditions such as education, employment and housing [12–13; 23]. Previous research has shown that housing problems such as damp/condensation, noise, cold and outstanding repairs explains a considerable amount of variance in occupants’ long-standing illness, self-assessed health, common symptoms, anxiety and depression [24]. Even when accounting for many other factors, poor housing conditions are significantly associated with children experiencing long-standing illness (particularly respiratory and digestive system problems), disability, smoking, drinking alcohol or the use of illegal drugs, with the duration of persistent poor housing increasing the likelihood of these outcomes further [25].

Kearns *et al*. [26] investigated the determinants of three psychosocial benefits of home which were identified through factor analysis of a large dataset from West Scotland: home as a haven (comprising privacy, the ability to get away, and safety); status (impact on self-esteem, safety, and control); and autonomy (acting as one likes and the feeling of being in control). After removing ‘general feelings’ from the model, housing problems had the greatest explanatory power of the variance of all three of the psychosocial benefits explored.

Housing improvements may therefore hold the potential for improving occupants’ health. A systematic review of 45 housing intervention studies concluded that there is good evidence for housing investments focussed on improving warmth having a positive impact on health, especially for older people and those in poor health [14]. Thomson *et al* [1] (pg62), based on a review of 39 housing intervention studies, concluded that ‘provision of adequate and affordable space and warmth are key determinants of subsequent health and health impacts, in particular respiratory health’.

Thomson and Thomas [13] (p210) produced an empirically-informed logic model for housing improvements which outlined the key outcomes as improvements in: ‘size and usable space; design; thermal comfort; costs (including fuel and rent); housing satisfaction and control over living environment; relationship with housing provider; and neighbourhood environment.’. Expansion in the amount of usable domestic space available to households through installation of central heating systems and other energy efficiency measures is especially key [1; 13]. This has been found to result in a greater number and variety of activities occurring within the home, including cooking, housework, homework, decorating and bathing [19–20]. Reductions in housing costs, as a result of housing improvement, increase disposable income [26] and have the potential to positively impact upon food choices and diet [13].

In addition to impacting upon these socioeconomic determinants of health, improvements may also hold the potential to improve the psychosocial benefits derived from home environments. Clark and Kearns [27] found that housing improvements have an indirect relationship with feelings of control and status derived from the home, via impacts on perceptions of home quality. The aspects of dwelling quality which were identified as contributing to psychosocial benefits included security of the home; electrical wiring; internal layout; overall space; heating system; internal decoration; quality or condition of bathroom/shower; and state of repair inside the home. Feelings of control were most strongly predicted by perceived security, whilst overall space within the home was the most important aspect of home quality for feelings of status [27].

Despite these potential benefits of home improvements, occupants’ experiences of housing interventions have not been universally positive. Some have reported a lack of control and sense of powerlessness at the time the improvement took place, due to interventions being provided free of charge rather than purchased privately [19–20]. It has also been shown that people can be confused and/or insufficiently informed regarding the use of new heating systems [19] and that warmth improvements do not necessarily result in energy savings [20]. Previous research also found a negative relationship between warmth/energy efficiency improvements and measures of perceived housing quality, although this shifted to a positive relationship when combined with other internal housing improvement measures [27].

Previous research has tended to focus upon evaluating the impact of one-off housing improvements facilitated through a particular scheme or initiative [19–20]. Individuals’ experience of accessing the improvement has therefore not been a primary focus of enquiry. This study instead augments the existing evidence base by exploring service users’ experiences of an information and advice team developed to facilitate health-focussed housing interventions for households on an ongoing basis as and when assistance is needed.

## Background to the Safe and Healthy Homes team

North Tyneside Council’s SHH Team was launched in September 2014 to provide an information, advice, support and guidance service to residents living in private rented or owner-occupied accommodation who have a housing issue which affected (or could affect) their physical or mental health. These occupants have historically received no or limited housing support from local authorities in comparison to those living in social rented properties. The team was managed by the Private Sector Housing Co-ordinator and an Innovation and Research Manager and consisted of two SHH officers who are Housing Health and Safety Rating System trained. The team receive direct referrals from residents and a variety of public, private and charity services. Individuals must be residents of North Tyneside and have a health issue or vulnerability that they perceive is being caused or exacerbated by their housing. If residents living in social housing contact SHH they are referred to the local authority’s property maintenance department or asked to contact their housing association.

After receiving a referral, an officer visits the client to complete an interview and an inspection of the property. Issues commonly faced by clients include excess cold, damp and/or mould growth, outstanding repairs, untidiness/clutter, trips and falls hazards and fire safety issues. SHH is not responsible for the direct provision of home improvements. Instead, the team provides information or refers clients to public, private and voluntary services which are able to help resolve their issues through housing interventions. The team does not have its own regular funding for capital investment in properties and so refers clients to funded services wherever possible but otherwise assists clients in choosing reliable and cost-effective traders. Officers also assist in facilitating the works by contacting companies on behalf of clients and attending when work is completed. For clients living in the private rented sector the team also contacts landlords to encourage them to make housing improvements to reduce or prevent negative impacts on their tenants’ health. Such cases are referred on to the Environmental Health team within the local authority if landlords fail to take action. Whilst the team focuses upon housing issues it also attempts to connect with wider public health agendas, providing information and making referrals for stop smoking, alcohol, drug, weight loss, physical activity and social isolation services. The officers also refer clients to benefit check services where appropriate.

Examples of the private and third sector services to which SHH refers clients include: a local charity which provide free draught proofing, energy efficiency advice, carbon monoxide detectors and welfare benefit checks to older people; a local not-for-profit organisation which can provide free or subsidised central heating systems, boilers, insulation and welfare benefit checks; and both private and not-for-profit tradespeople who can carry out repairs and complete work to reduce falls hazards. SHH also refer clients to public sector services such as: the local fire service which provide smoke detectors and fire safety advice; the minor aids and adaptations service within the local authority which fits banisters and bathroom grab rails without charge; and a team within the local authority’s adult social care service which assists people suffering from social isolation to connect with local activity groups. As well as acting as outgoing referral destinations for clients, many of these services also act as referral routes into SHH. Services often refer clients to SHH such that assistance can be sourced for housing issues which are outside of the referring services’ remit.

## Methods

The aim of the study was to explore the experiences and self-perceived health and wellbeing outcomes of clients who have used North Tyneside Council’s SHH service. This aim was addressed by adopting a qualitative methodology to provide an in-depth understanding of the participants’ experiences and outcomes of their use of the SHH service. The flexible and open-ended nature of qualitative research enabled the participants to explore the meaning that they attached to their experience in addition to exploration of their views of the housing interventions that they received from the service during an in-depth interview [28–29].

### Sampling strategy and sample

A purposive sampling strategy was adopted to identify individuals with specific knowledge and situational prerequisites that directly related to the research [30]. This approach also scopes for diversity within a population so that breadth of experience of the phenomenon can be maximised. Potential participants were identified from the service’s database and were only included if they had been referred within the last 12 months and if at least one intervention had taken place. Information on the health conditions of household members and the scale of intervention received was used in order to produce a mixed sample of service users.

Though there are different views about the optimum sample size in qualitative research, varying from 6–20 participants, there is general agreement that ‘richness’ should be the overriding consideration in determining sufficiency of data [31–32]. Fifteen households were recruited to the study from a total of 538 which had been referred in the previous 12 months. The ages of the lead client of the households ranged from 47 to 84 with a median age of 62. Nine were female and six were male. 14 of the householders were owner occupiers and one householder privately rented their property (Household 4 in Table 1). Members of the households suffered from a range of chronic diseases and comorbidities. Further details of the householders are presented in Table 1.

**Table 1:** Participating households.

Household	Household Size	Length of Residence in Property	Referral Route to SHH	Household Health Issue Classification	Outcomes Achieved
1	2	38 years	GP Surgery	Musculoskeletal; respiratory; cardiovascular; renal.	Draught excluders installed; second banister installed; grab rails installed in bathroom; central heating serviced and thermostat moved; assistance completing benefit application
2	2	12 years	GP Surgery	Mental health; respiratory; musculoskeletal.	New boiler installed; benefits check completed; draught proofing completed upstairs; second banister installed; grab rail installed in bathroom; tapturners installed; walking sticks provided for upstairs; kitchen assessment completed
3	4 (2 children)	16 years	Self-referral	Mental health; respiratory; hearing.	New boiler installed
4	1	56 years	Heating charity	Mental health	Two banisters installed
5	3 (2 children)	26 years	Self-referral	Musculoskeletal	New boiler installed; second banister installed; smoke alarms fitted and fire safety check
6	2	41 years	Self-referral	Musculoskeletal; respiratory	New central heating system installed; window repaired
7	2	36 years	Heating charity	Musculoskeletal	New letterbox fitted to prevent draughts; emergency tiling completed on roof; pointing work completed
8	2	44 years	Self-referral	Musculoskeletal; cardiovascular	New boiler installed; second banister installed; grab rails installed in the bathroom; draught proofing completed
9	1	Nearly 30 years	Local authority Environmental Health team	Musculoskeletal	New boiler installed
10	2	28 years	Self-referral	Respiratory; cardiovascular; musculoskeletal.	Under-floor insulation installed; leaking tap replaced
11	2	48 years	Heating charity	Musculoskeletal; previously cancer	Draught proofing completed; carbon monoxide detector installed
12	4 (1 child)	Nearly 19 years	Self-referral	Respiratory	New central heating system installed; new electricity meter, earth wire and switches installed
13	3	36 years	Older people’s charity	Metabolic	Second banister installed
14	2	49 years	Self-referral	Respiratory; cardiovascular; metabolic; renal; inflammatory and immune system; musculoskeletal; metabolic.	New boiler and four new radiators installed; new banister installed; grab rail installed in bathroom; rail installed in bedroom; food and drink trolley provided; ‘Care Call’ pendant system installed
15	1	9 years	Local authority’s social isolation team	Respiratory; renal	New boiler and radiator installed and fire removed; security light repaired

### Data collection

In-depth, semi-structured interviews were completed in the participants’ own home during 2016. This provided an opportunity for the participant to show the researcher the work and adaptations that had been completed, and this stimulated detailed discussion of the difficulties that they had experienced with the design, other features of their home, and how the housing interventions had affected their life. All interviews were digitally recorded and transcribed verbatim in preparation for analysis. The participants were asked to discuss the details of their tenancy, the housing intervention they had received and their health conditions, before exploring more abstract questions on the relationship between housing and health, how their housing affected their health and personal wellbeing, their experience of the housing service, and how the housing intervention affected their health, wellbeing and lifestyle.

### Analysis

The transcripts were read, re-read and independently open coded by individual members of the research team. Randomly selected transcripts were independently coded by another team member, and the outcomes were compared with the original coding to validate findings. This allowed elucidation and description of participants’ experiences of the housing services and related interventions whilst creating meaningful themes [33]. Emerging codes were discussed amongst the research team, allowing for greater reflection and the ability to move between the data and findings.

Whilst this study is relatively small-scale, it has advantages and novelties over previous service developments and related housing improvements, and wellbeing studies. First, the SHH team focuses its service on residents in poor health, in contrast to some previous research which has simply focussed on housing improvements without regard to individuals’ original level of health [19–20; 28]. Secondly, the SHH team delivers its service to people in private accommodation, whereas previous studies have often focussed upon social housing [19; 28].

## Ethical considerations

Research ethics approval to carry out the study was provided by the Faculty of Health and Life Sciences, Northumbria University. Participants were informed about the aims of the study, signed a consent form, and participated voluntarily. All data were anonymised.

### Reflexivity

The research was conducted as part of a series of research projects considering the relationship between housing and health, with several completed with support from North Tyneside Council. The team comprised researchers with backgrounds in nursing, older people, healthcare, social science and housing.

## Results

The participants were acutely aware that their home environment had a marked impact on their everyday experience. This impact was positive when the house, its design and internal fittings were adapted to the changing needs of the occupants. Conversely, when the participants did not know what adaptations or improvements could be made to their home, or how to make or fund those changes, they suggested that their daily life was difficult. The findings commence with the participants’ views that the compatibility of their personal needs (and those of other home occupants) and home environment had decreased; this is followed by their views of their experience of the housing intervention; and the final theme presents the participants’ views of the impact of the housing services on their health and wellbeing.

### Theme One: Degenerating person-home fit

The participants, or members of their family, had experienced health conditions that may have contributed to disability and/or mobility problems. Whilst, for some, deterioration in their abilities had developed over a long period of time, for others changes in personal abilities had occurred as a result of an acute illness, injury or surgery. Many discussed mobility problems and how they were no longer able to use the fixtures and fittings of their home:

I can get in the bath but I can’t get out…if I had a bath I wouldn’t be able to get out…I can’t lever myself up to get out

(Household 8, 2 individuals, 44 years at the residence)

These individuals were acutely aware of the hazards associated with using their bathing fittings and how limited bathing affected their personal hygiene. Some participants restricted their interaction with other people as a consequence of embarrassment about their hygiene, thus becoming more socially isolated. Difficulty using stairs was regularly mentioned by the participants:

I had a knee replacement and I had difficulty getting up and down the stairs, even after I had the knee replacement.

(Household 8, two individuals, 44 years at the residence)

The difficulty and pain associated with climbing stairs influenced decisions to restrict movement and as a consequence they largely lived in one part of their house. Decisions about leaving the home were also carefully deliberated. They highlighted the mismatch between their housing condition and what they wanted to do. This affected their mental wellbeing:

Oh, I'm usually always happy. It's just because I couldn't provide heat and hot water for [my children], I just got agitated and…upset.

(Household 5, three individuals, 26 years at the property)

The negative feelings expressed by this participant represented the views of others in depicting stress and anxiety of daily life. This often contributed to ‘*snapping at each other’*, irritation and arguments between occupants. Yet there was much contradiction in the discussions when the majority of participants also spoke of their home with warmth and affection. This was the place where they had brought up their family and had lived their life. It was a place that was associated with many memories. It continued to be a place where they knew and were supported by their neighbours, they felt safe and secure, and enjoyed a lifestyle that was supported by local services and participation in community events.

The participants also spoke of their increasing limitation to do housework. This contributed to deterioration of their home:

And I’ve had about 13 operations…Because I can’t get my housework done when I want to. I’ve got to wait until I feel fit enough. And I get halfway through it and I’ve got to stop. At one time, I used to rush through, vacuuming upstairs, vacuuming downstairs. Now, one room–and I’m no good. So what used to take me a couple of days to do the house is taking me a fortnight.

(Household 14, two individuals, 49 years at the residence)

The experience of not being able to undertake what would have been routine activities and simple repairs at a different stage in life reinforced their perception that they were losing independence:

Well, yeah, just the other day the sink was blocked. And I’m like, right… And I couldn’t get under the sink to unscrew the tap. So it makes you feel very dependent on others, which I don’t like.

(Household 2, two individuals, 12 years at the residence)

Living on a fixed income was a major concern for many individuals. They were acutely aware that repairs and maintenance of their home was important because deterioration in their housing would have a negative impact on their health. Yet financing repairs involved difficult choices, sometimes between eating and heating. The deteriorating condition of their home, ‘*stuff breaking down*,*’* alongside inability to finance the needed repairs certainly took its toll on individuals who already anticipated a future of decline and disability:

Just the added stress of stuff that you think, oh god, what am I going to do now? Have I got to live like this forever? What if I have to stay downstairs all the time? Or what if I sat upstairs all the time?…It was very much a…What’s going to happen next? How will I get on?

(Household 2, 2 individuals, 12 years at the residence)

### Theme Two: Knowledge of and gaining access to housing and other support services through Safe and Healthy Homes

The participants were aware of their housing problems, and some could identify potential solutions, yet they lacked knowledge or experience of local services, organisations and traders who could assist with rectifying housing issues:

You see things in people’s houses, like my neighbour down the road, he’s got two stair banisters but you don’t think about how it was done.

(Household 1, two individuals, 38 years at the residence)

Others commented on how they were reluctant to find out about and use services and organisations because of poor previous experiences. Some had previously applied for grants for housing improvements or use of services to find that they were not eligible. Consequently, they tolerated their circumstances:

I think the problem is we’ve never asked anybody for anything. We’ve just sort of mosied on and got on with it. And if something has gone wrong, it’s had to just stay wrong until we’ve had the coppers to [fix it].

(Household 7, two individuals, 36 years at the residence)

The participants had little previous experience of housing services. They placed great importance upon the personal interaction with SHH officers. They valued the officers’ personal qualities and described them as empathetic, non-judgmental, empowering, effective communicators, good listeners, competent, supportive and helpful. They suggested that this enabled them to talk about difficulties and sensitive issues. For several participants the personal interaction with the officer was central to their experience:

[The officer] sat there…absolutely lovely, listened, you felt the compassion…and she says as soon as she walked in the door she thought ‘she needs a second banister’. Soon as she walked in the door she knew there were difficulties and she listened.

(Household 1, two individuals, 38 years at the residence)

For one interviewee the first home visit provided a sense of validation of her health conditions and housing problems that she had not received from other professionals:

I think that I was going [to my doctor] before because I probably needed the support, more than anything else. My knees [would] be sore–send you for an X-ray…You didn’t get the validation. And that’s one of the major things that started with the very first visit. So I think when someone has come out to your house and said, ‘Yes, you need that help, we will do it,’ it’s such an affirmation.

(Household 2, two individuals, 12 years at the residence)

Interviewees described how the SHH officers recognized that they did not know who or what services to contact in order to address their problems. They appreciated the way the officers made suggestions for them to consider and then connected them through referral or by providing contact details of various services, organisations and independent traders:

Well [the officer] put us through different places for grants …he sorts like everything out for us like the light outside and Warmzone. I got the walk in shower put in…[the officer] got on to the occupational therapist for us and suggested ‘a rail going up the stairs might be helpful’…[the officer’s] really helpful, he gives you all the details and contact numbers, everything…to get you in touch [with traders]…if I’m worried about something I’ve got his contact number straight direct to him and I phone him… I didn’t know where to turn, I needed help… I was in rock bottom…until I went [to the officer] and ever since he’s been good.

(Household 15, 1 individual, nine years at the residence)

The team’s assistance with sourcing funding for work or receiving a benefit check that might lead to increased income was highlighted by some participants. The officers worked with clients to find out how their needs could be addressed by professionals and teams across sector and service boundaries:

[The officer] said ‘look you can’t put up with…the radiators and what have you, I’ll set the wheels in motion’ then she said ‘was he in the forces?’ and when he said yes…it was all down to [the officer], she said ‘I’ll get in touch with SSAFA [Armed Forces charity]’ and then SSAFA rang.

(Household 6, two individuals, 41 years at the residence)

Some participants made it clear that even though SHH officers took a large role in facilitating housing interventions, they still had ultimate choice and control over the work completed:

it’s up to yourself, if you want to ring…say like I wanted this place decorated, it’s like [an] estimate, [the officer] would get on to them and they would phone me and they would write me an estimate and it’s up to me to say ‘that’s too much I cannot afford that.’

(Household 15, 1 individual, nine years at the residence)

Many interviewees spoke of how there had been continued interaction with officers after the initial visit regarding forthcoming work and offers of further help after the interventions had been delivered. Participants discussed how they were reassured to know that they now had a contact who knew how the system works and what services are available. Importantly the officer would be able to assist them in the future should they need any advice concerning their housing and other issues:

I’ve got [the officer’s] number…I’m sure she would help if I said well ‘would we be eligible for another benefit or some help towards this that or the other’ and she would point us in the right direction or she would do it for us.

(Household 11, two individuals, 48 years at the residence)

Other participants were pleased to have been made aware of the services to which SHH had connected them, such as SSAFA and benefits advisors. However, one participant reported feeling as though the follow up from SHH could be improved and suggested that officers revisit clients at their home one year later to offer further help.

### Theme Three: Perceived health and wellbeing outcomes of the housing interventions

The participants were predominantly positive about the service, both in terms of the outcomes of the interventions and their interaction with the officers. The reported benefits were therefore unsurprisingly varied, ranging from specific outcomes such as improved independence when addressing personal needs (such as bathing without assistance), increased mobility in the home (particularly climbing stairs), less acute exacerbations of respiratory problems, improved interpersonal relationships between occupants, reduced expense in maintaining household facilities within the home, to broader discussions of feeling happier, more confident and less stressed. They attributed these outcomes directly to the interventions received from housing services:

with two banisters on the stairs it’s much better especially when you’re carrying clothes or whatever up the stairs. I can carry stuff up and down it’s great so it’s helped me to get my life back. It’s the same with the bath I just hold the little handrail and get out safely. In the toilet, I just pull myself straight up. Its great so it’s made a big difference to my life.

(Household 1, two individuals, 38 years at the residence)

The most challenging need for support came from problems resulting from changes in personal circumstances such as worsening health conditions or new faults in household equipment. These brought multiple challenges, as not only did participants speak of the need to address these issues but also the difficulties they created in attempts to live normally at home. As such, problems were exacerbated by the physical and mental strain deriving from having to adapt taken-for-granted behaviours to overcome these problems:

When you're in your own home you want to have a hot bath or shower. Well with no hot water at all, I couldn't even do dishes. I had to boil kettles. And it was absolutely awful…and now I can do everything. I feel so much better.

(Household 5, three individuals, 26 years at the residence)

Therefore, the outcomes supported by the officers, such as new boilers and heating systems, not only led to perceived improvements in living conditions in terms of temperature and warmth, whilst simultaneously restoring basic facilities like hot water, but also reportedly increased the mood and wellbeing of the participants.

It’s taken the worry away about should we have the heating on less or sit with me coat on or whatever, you don’t worry about that at all put the heating … it’s not a worry, we don’t worry about the cost any more where in the past we’d be thinking…you know gas bills go right up or electricity bills going up.

(Household 1, two individuals, 38 years at the residence)

This support therefore enabled participants to overcome changes in the home environment, whilst simultaneously underpinning feelings of comfort and perceived increases to wellbeing in relation to living and behaving at home as they choose.

However, these services also brought perceived benefits, which extended beyond the home environment. Participants identified a range of wider potential changes to their circumstances, including fewer GP appointments, reduced stress, increased access to services and improved personal health conditions.

Well, since the house has been warm, he’s breathing a lot better. He’s not coughing his lungs up as much … But with the rooms being warm, he’s not having to put a Calor gas or anything on and cough his lungs up. So it’s [been] better for him as well. It’s helped him a lot.

(Household 12, four individuals, nearly 19 years at the residence)

One recurring potential benefit for other services was the increased safety participants felt in their own home, which was seen as particularly likely to reduce falls.

It makes it possible for me, now, to not fear the stairs, which I did. You know? And underneath it was a fear of falling … So the grab rails were a great benefit to me, and just mentally, even if nothing else. But physically it makes it a lot easier for me to handle that. Amazing–a bit of wood.

(Household 2, two individuals, 12 years at the residence)

Reported negative outcomes were rare, and only arose when participants felt their needs were not being specifically addressed, either as a result of changes to the home which were not deemed as a priority or perceived as aesthetically unappealing. This was best represented when one participant was given tap turners which were perceived to be lacking practical use, out of step with the design of the house and disempowering.

Not like the tap turners has affected the aesthetics here. Oh, they were awful. Seriously … Well, you see what I mean? I mean, you don’t want to…wave a flag and go, ‘Oh, I’m disabled, you know.’

(Household 2, two individuals, 12 years at the residence)

Thus, while many of the outcomes of the interventions were seen as immediately beneficial by and for the participants, these impacts were not limited to improving the physical environment of the house. They also brought additional perceived benefits in terms of wellbeing and confidence, as long as the changes that were made were seen as reinforcing the participants’ own views of how their homes should be supportive of their specific needs and activities.

## Discussion

The participants generally suffered from chronic health conditions which were exacerbated by the hazards in their housing environment, causing considerable stress and anxiety. Interviewees reported that the housing interventions facilitated by the SHH team were able to improve their living conditions which they perceived as positively impacting upon their personal health and wellbeing, including feelings of comfort and a sense of independence and security. These findings therefore corroborate some of the previous research into the impact of housing improvements on health and wellbeing [19–20; 26; 27]. However, the study augments these findings by exploring the operation of a service which is geared towards ongoing support and advice rather than one-off housing improvements. The primary contribution of this research is the demonstration of the emphasis clients placed upon interaction with the SHH officers who were able to connect them to a network of professionals and services that could provide assistance or home improvements both when the initial need arose and for the future.

Clients of the SHH team live in private sector accommodation and are perhaps less likely to have a history of using state and charitable services. Participants were generally unaware of how they might access support in relation to their housing, did not have an understanding of how services operated and lacked ‘system trust’ in the willingness of the welfare state (in its widest sense) and others to help them [22,34]. The findings highlight how the SHH officers formed an ‘access point’ [22] into the complex system of the modern welfare state and its associated network of private and third sector organisations which can provide assistance. Clients place great value upon the ‘facework’ in which the team engage which forms an important opportunity to engender trust in the ability of both the officers and the wider system to assist them [22]. SHH provides clients with a trusted access point into a wider system of assistance built upon direct interpersonal contact. These participants valued being able to talk with the officers about their housing-related problems and being navigated through systems of which they were either unaware or perceived to be very unresponsive and complex. The study demonstrates that the existence of health-focussed housing interventions alone can be insufficient to achieving positive health and wellbeing outcomes; individuals must understand how to navigate this system in addition to it simply being available. Without the potential for facework-dependent access points into such support systems, the most vulnerable people may be unwilling to engage with a confusing network of services of which they either know little or have had negative prior experiences, allowing their health, wellbeing and ontological security to decline further. Appreciation of this will be critical to ensuring that future support services are successful as the UK and other countries continue to respond to the policy challenge of poor housing and its impacts on health and wellbeing.

However, the participants’ experiences of benefits beyond their expectations and knowledge of existing systems highlights a potential for the importance of facework to be reciprocal here. Just as the participants used the officers as access points to the systems, the systems themselves used the officers as access points to all the inherent complexity found within this population. Without the relationship formed between both parties, it would have been unlikely that the service would have been aware of the often subconscious needs of the participants, none of whom contacted the service with the goal of improving their access to services or to reduce GP appointments, for example. Yet, with the access points created, these benefits can be achieved. Thus, creating this knowledge of the person within, and for, the system allows a range of services to meet the needs of people more effectively. This is especially valuable for the British welfare state, which requires increasing integration of housing, health and adult social care services and is increasingly reliant upon the private and voluntary sectors. As such, the benefits of facework sit centrally in the relationship between the complexity of needs of both service users and the multiple systems that must meet them.

There are several limitations to the findings of this study that should be noted. First, the sample of clients is very limited in size and is taken from just one health-focussed housing team. This raises the potential that the experiences explored here are unrepresentative of both SHH and such services more generally. The findings produced therefore need to be treated with some caution and generalisation of the findings across SHH and to clients of similar services should be avoided. Secondly, interviews with participants were only completed after interventions had taken place. Reports of impacts on health and wellbeing therefore rely upon resident recall, rather than standardised measures of changes. The apparent benefits of the service are as perceived and reported by participants rather than via objective analysis. Thirdly, the research used a qualitative methodology and therefore did not include a control group within the study. Without a comparison group, it is possible that the participants may have discovered and made use of appropriate services without the guidance and support of SHH. Future research should consider the different approaches taken by such guidance services within local authorities and how they ensure that residents are aware of and use these access points.
